# Smoothing Effect in Vital Sign Recordings: Fact or Fiction? A Retrospective Cohort Analysis of Manual and Continuous Vital Sign Measurements to Assess Data Smoothing in Postoperative Care

**DOI:** 10.1213/ANE.0000000000003694

**Published:** 2018-08-09

**Authors:** Hamish R. Tomlinson, Marco A. F. Pimentel, Stephen Gerry, David A. Clifton, Lionel Tarassenko, Peter J. Watkinson

**Affiliations:** From the * Department of Engineering Science, Institute of Biomedical Engineering, University of Oxford, Oxford, United Kingdom; † Centre for Statistics in Medicine, Nuffield Department of Orthopaedics, Rheumatology and Musculoskeletal Sciences, Botnar Research Centre, University of Oxford, Oxford, United Kingdom; ‡ Kadoorie Centre for Critical Care Research and Education, Nuffield Department of Clinical Neurosciences, Oxford University Hospitals National Health Service (NHS) Trust, Oxford, United Kingdom.

## Abstract

**BACKGROUND:**

Data smoothing of vital signs has been reported in the anesthesia literature, suggesting that clinical staff are biased toward measurements of normal physiology. However, these findings may be partially explained by clinicians interpolating spurious values from noisy signals and by the undersampling of physiological changes by infrequent manual observations. We explored the phenomenon of data smoothing using a method robust to these effects in a large postoperative dataset including respiratory rate, heart rate, and oxygen saturation (Spo_2_). We also assessed whether the presence of the vital sign taker creates an arousal effect.

**METHODS:**

Study data came from a UK upper gastrointestinal postoperative ward (May 2009 to December 2013). We compared manually recorded vital sign data with contemporaneous continuous data recorded from monitoring equipment. We proposed that data smoothing increases differences between vital sign sources as vital sign abnormality increases. The primary assessment method was a mixed-effects model relating continuous-manual differences to vital sign values, adjusting for repeated measurements. We tested the regression slope significance and predicted the continuous-manual difference at clinically important vital sign values. We calculated limits of agreement (LoA) between vital sign sources using the Bland–Altman method, adjusting for repeated measures. Similarly, we assessed whether the vital sign taker affected vital signs, comparing continuous data before and during manual recording.

**RESULTS:**

From 407 study patients, 271 had contemporaneous continuous and manual recordings, allowing 3740 respiratory rate, 3844 heart rate, and 3896 Spo_2_ paired measurements for analysis. For the model relating continuous-manual differences to continuous-manual average vital sign values, the regression slope (95% confidence interval) was 0.04 (−0.01 to 0.10; *P* = .11) for respiratory rate, 0.04 (−0.01 to 0.09; *P* = .11) for heart rate, and 0.10 (0.07–0.14; *P* < .001) for Spo_2_. For Spo_2_ measurements of 91%, the model predicted a continuous-manual difference (95% confidence interval) of −0.88% (−1.17% to −0.60%). The bias (LoA) between measurement sources was −0.74 (−7.80 to 6.32) breaths/min for respiratory rate, −1.13 (−17.4 to 15.1) beats/min for heart rate, and −0.25% (−3.35% to 2.84%) for Spo_2_. The bias (LoA) between continuous data before and during manual observation was −0.57 (−5.63 to 4.48) breaths/min for respiratory rate, −0.71 (−10.2 to 8.73) beats/min for heart rate, and −0.07% (−2.67% to 2.54%) for Spo_2_.

**CONCLUSIONS:**

We found no evidence of data smoothing for heart rate and respiratory rate measurements. Although an effect exists for Spo_2_ measurements, it was not clinically significant. The wide LoAs between continuous and manually recorded vital signs would commonly result in different early warning scores, impacting clinical care. There was no evidence of an arousal effect caused by the vital sign taker.

**KEY POINTS**

- **Question:** Does a “smoothing effect” exist between manually recorded and electronically recorded vital sign measurements in postoperative care?
- **Findings:** Using a mixed-effects model, we found no relationship between continuous-manual differences and continuous-manual average values for heart rate and respiratory rate, and we found a weak (but clinically insignificant) relationship for oxygen saturation.
- **Meaning:** We found that clinical staff in a postoperative ward did not “smooth” vital sign values with a bias toward recording more normal readings because the differences between manual and continuous vital sign measurements were not related to the vital sign values.

Manual measurements of the main vital signs—which include respiratory rate, blood pressure, heart rate, temperature, and oxygen saturation (Spo_2_)—are often inaccurate.^1–3^ Manual calculations of clinical risk scores are also error prone.^4–6^ While automated monitoring technology exists, it is mostly confined to high-acuity patients, and manual measurement and documentation of vital signs remain the standard of care in many wards. A potential source of inaccuracy that may exist in the manual vital sign record is data smoothing.^7^ Clinical staff may be biased toward vital sign values that lie within the assumed limits of normality and record vital sign values that are incorrectly normal— “smoothing” the extremes in the vital sign record. If real, this “smoothing effect”^8^ may result in lost opportunities for early recognition of physiological deterioration.

The smoothing effect has been reported to occur during anesthesia^8–13^ and in acute ward monitoring.^14,15^ Most studies use methods based on the comparison of vital sign values from manual and automated measurements sources.^9–15^ The comparison method makes the cause of “data smoothing” unclear. Vital sign values from monitoring equipment are noisy and may be corrupted with signal artefact, so the smoothing effect may partly result from clinicians correcting spurious values.^14^ Undersampling may also affect data smoothing based on the magnitude and frequency of extremal values in longitudinal records because sparsely sampled manual observations may not coincide with times of large fluctuations in vital sign values. Analysis compensating for these confounding factors is essential to discover whether the smoothing effect is of clinical relevance.

We present a secondary analysis of a large database of postoperative vital sign records to investigate data smoothing of respiratory rate, heart rate, and Spo_2_. We propose that data smoothing increases the differences between continuous and manual vital sign measurements as the (absolute) value of the vital sign becomes more extreme. We tested whether differences between continuous and manual vital sign recordings are related to the average value of the 2 vital sign recordings. We also assessed agreement between continuous and manual data. Finally, we investigated whether there is an arousal effect caused by the vital sign taker.^15^

## METHODS

This article adheres to the Reporting of Studies Conducted Using Observational Routinely-Collected Health Data statement, an extension of the Strengthening the Reporting of Observational Studies in Epidemiology guidelines.^16,17^

### Dataset

The database for this retrospective analysis was created during the Computer Alerting Monitoring System 2 (CALMS-2) study, which was granted ethical approval (Mid and South Buckinghamshire ethics committee Research Ethics Committee: 08/H0604/79, December 9, 2008, and Leeds [West] ethics committee Research Ethics Committee: 11/YH/0056, May 20, 2011) and registered in the International Standard Randomised Controlled Trial Number (ISRCTN) database (principal investigator: P.J.W., ISRCTN No: ISRCTN58660550, August 11, 2017). This study assessed whether ambulatory physiological monitoring combined with an alerting system improved recognition and outcomes in patients after major surgery.

Vital sign data used in the CALMS-2 study were collected in a step-down postoperative ward of the Oxford University Hospitals National Health Service (NHS) Trust, Oxford, between May 2009 and December 2013. Potential participants were screened during preoperative assessment and deemed eligible if they were planned to undergo major upper gastrointestinal surgery. This category was defined as follows: oesophagectomy, oesophagogastrectomy, gastrectomy, Whipple’s operation, liver resection, pancreatectomy, gastric bypass, biliary reconstruction, and splenectomy. Participants were excluded based on the following criteria: participants <16 years of age, pregnant women, participants unable to wear the required monitoring, participants without the capacity to consent, and participants who could not understand written English and for whom no translator could be found. For this secondary analysis, patients who did not receive contemporaneous bedside electronic vital sign monitoring and manual vital sign observations were excluded a priori. Written informed consent was obtained for all subjects.

In the step-down ward used in the CALMS-2 study, high-risk patients are admitted after a period of elective intensive care unit stay, while patients with a lower risk of complication are admitted to the ward immediately after surgery. High-risk patients typically receive an increased level of care for the first 2–48 hours of their ward stay, during which time they undergo conventional bedside monitoring, consisting of continuously measured respiratory rate, heart rate, and Spo_2_ (Philips M3046A/Intellivue MP50 clinical monitor; Philips Healthcare, Best, the Netherlands). Respiratory rate was measured by impedance pneumography, heart rate was derived from the electrocardiogram, and Spo_2_ was measured by pulse oximetry. Clinical staff also made manual measurements of blood pressure and temperature, typically at hourly intervals. After this initial period, these patients then join the other patients on the ward in receiving general-ward-level care, which consists of manual vital sign recording typically at 4-hour intervals. The standard of care for measuring respiratory rate on the ward is by counting chest wall movements, and heart rate and Spo_2_ measurements were likely to be transposed from the monitor screen. In the CALMS-2 study, ambulatory monitoring of heart rate and Spo_2_ was undertaken. However, for consistency, we restricted this analysis to manually recorded values that could be compared with contemporaneous values from bedside monitoring.

Manual vital sign measurements documented on paper-based bedside charts were double entered into an electronic database. A third researcher reconciled differences with access to the original charts.^5^ Continuous vital sign data from the bedside monitors were saved directly from the monitor every second. The final dataset obtained for this study consisted of vital sign records of respiratory rate, heart rate, and Spo_2_ from continuous bedside monitoring equipment and manual vital sign observations.

Manual and continuous vital sign values were compared based on the timestamps taken from paper records and the computer-generated timestamps from the patient monitor. Clinical staff manually recorded blood pressure measurements at the time of observation, and these were also logged in the automatically generated data record. We set the manual observation time for all vital signs to the computer-generated timestamps for blood pressure (while checking that data were correctly matched). This calibration method ensured that the data considered contemporaneous from manual and continuous measurement sources were synchronized, and thus could appropriately be used for comparison.

### Statistical Analysis

We summarized the number of vital sign measurements included per patient using the sample median and interquartile range.

We sampled the continuous data at the time of each manual observation to create paired measurements of continuous and manual vital signs. We sampled the continuous data by extracting the median value of a 5-minute window centered at the time of manual observation. We used this methodology (also known as “median filtering”) to summarize the continuous data without the effects of measurement noise or short-term variance while retaining long-term vital sign trends. We included all manual observations with contemporaneous periods of continuous data, allowing patients to provide multiple observations in our analysis. We selected a 5-minute window to reflect clinical practice, in line with previous work.^13,15^ We undertook sensitivity analyses by recomputing the primary assessment method for windows of 1–10 minutes.

To obtain the differences between the 2 measurement sources, we subtracted the manual vital sign from the continuous vital sign in each measurement pair. We also calculated the average of the continuous and manual vital signs for each measurement pair. We modeled the relationship between the differences and the averages of the measurement recordings using a linear mixed-effects model, with averages as a fixed effect and subject as a random effect to adjust for repeated measurements. We note that there may be factors other than a smoothing effect that could affect the relationship between the continuous-manual difference and the continuous-manual average, notably the accuracy of the continuous monitoring equipment. However, given our use of gold standard monitoring equipment and median filtering to remove transient outliers, the continuous data were likely to be equally reliable over the range of measurement values. Thus, a linear relationship between the continuous-manual difference and the continuous-manual average would be best explained by a clinical bias in the manual data, allowing the smoothing effect to be tested.

The regression slope of the fixed effect in the mixed-effects model represents the increase in the continuous-manual difference for a 1-unit increase in the continuous-manual average. Statistical significance of the regression slope was calculated with an *F* test (type III with Kenward–Roger degrees of freedom approximation),^18^ using a significance of 0.05 to reject the null hypothesis that the regression slope was 0 (no relationship). To assess the clinical significance of the linear relationship, we simulated the mean differences and 95% confidence intervals (CIs) predicted by our model at vital sign values corresponding to important clinical thresholds. We used respiratory rate values of 8 and 24 breaths/min, heart rate values of 40 and 130 beats/min, and an Spo_2_ value of 91%, which are the maximum National Early Warning Score limits for each vital sign.^19^

We generated Bland–Altman plots for the continuous and manual vital sign data by plotting the differences between the 2 measures (y-axis) against the averages of the 2 measures (x-axis). The bias was calculated as the mean difference between continuous and manual measurement pairs. Limits of agreement (LoAs) were calculated using a mixed-effects model, including a subject random effect to adjust for repeated measurements.^20^ CIs (95%) were calculated for the bias and the LoAs using the method recommended by Bland and Altman.^21^ Horizontal lines were included on the Bland–Altman plots to show the bias and LoAs. The regression line from the linear mixed-effect model was plotted to visualize the relationship between the differences and the averages.

**Figure 1.**
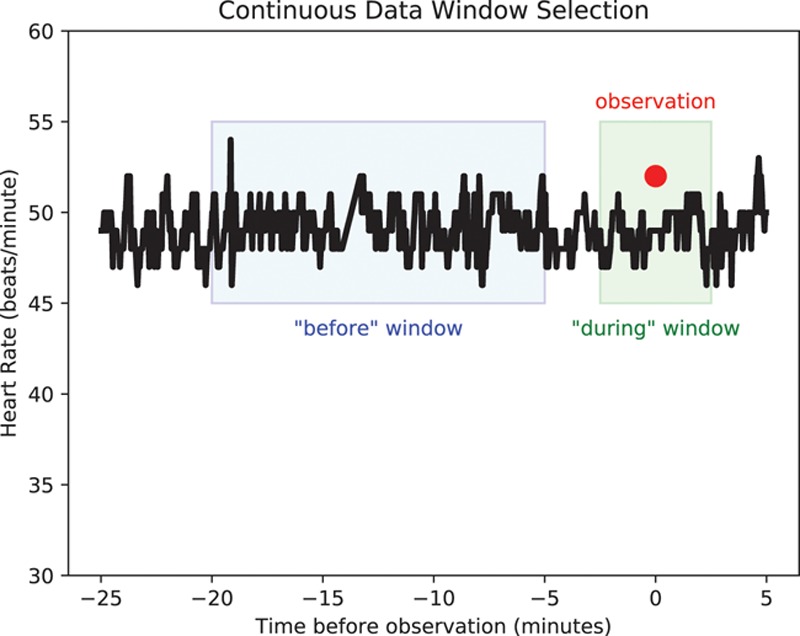
An example heart rate observation, for which the “before” window is shown in blue, the “during” window is shown in green, and the manual observation (synchronized to the time of the blood pressure measurement) is shown in red.

We assessed whether there is an arousal effect caused by the vital sign taker by comparing continuous data before and during the time of manual observation. For the continuous data before the observation, we used the median value of a 15-minute window ending 5 minutes before the time of observation. For the continuous data during the time of observation, we used the median of a 5-minute window, as described in previous paragraphs. These methods replicate those of Taenzer et al,^15^ to allow comparison. The selection of the continuous data is shown schematically for heart rate in Figure 1. We prepared Bland–Altman plots for the “before” and “during” measurements using the same methodology described in the previous paragraph.

We did not perform sample size calculations for this study because it is a retrospective cohort study of an existing dataset—we used all available data, noting that our dataset was larger than those used in most previous analyses of the smoothing effect.^9–13,15,22^ We also could not identify the statistical power of the study since previous studies of “the smoothing effect” have not used Bland–Altman analyses (preventing estimation of approximate population regression slopes). We instead provided CIs for all reported outcomes to demonstrate the precision of our results, as recommended by Goodman and Berlin.^23^

## RESULTS

**Figure 2.**
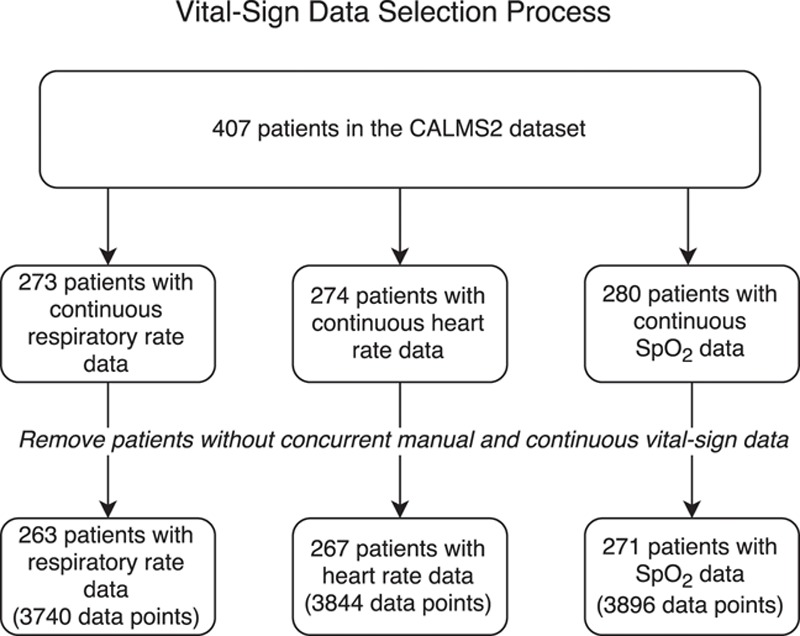
The vital sign data selection process. CALMS-2 indicates Computer Alerting Monitoring System 2; Spo_2_, oxygen saturation.

Patient inclusion is shown in Figure 2. Concurrent manual and continuous vital sign measurements were available for respiratory rate from 263 patients (3740 paired measurements), heart rate from 267 patients (3844 paired measurements), and Spo_2_ from 271 patients (3896 paired measurements). The median (interquartile range) number of observations for each patient was 11 (7–18) respiratory rate, 11 (7–18) heart rate, and 11 (7–19) Spo_2_ measurements.

**Figure 3.**
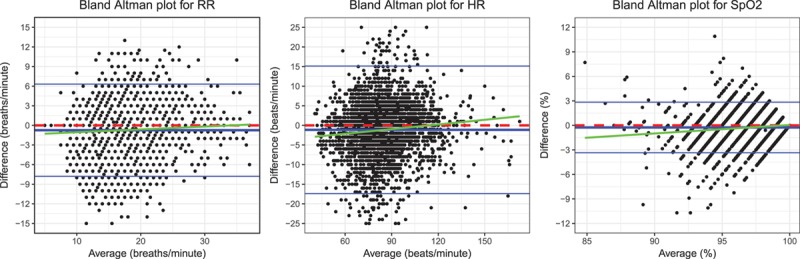
Bland–Altman plots for RR, HR, and Spo_2_ showing limits of agreement between the continuous data and the manual observation (continuous data–manual observation). Bias and limits of agreement are shown with blue lines, the regression line is shown in green, and a dashed red line shows the zero y-intercept. HR indicates heart rate; RR, respiratory rate; Spo_2_, oxygen saturation.

The mixed-effect model regression slope (95% CI) between the continuous-manual difference and the continuous-manual average was 0.04 (−0.01 to 0.10; *P* = .11) for respiratory rate, 0.04 (−0.01 to 0.09; *P* = .11) for heart rate, and 0.10 (0.07–0.14; *P* < .001) for Spo_2_ (Figure 3 and Table). The mean differences (95% CI) predicted by the model were −1.14 (−1.57 to −0.71) and −0.44 (−1.57 to 0.04) breaths/min for respiratory rates of 8 and 24 breaths/min, respectively. Likewise, the differences were −2.93 (−4.75 to −1.11) and 0.63 (−4.75 to 3.26) beats/min for heart rates of 40 and 130 beats/min, respectively, and −0.88% (−1.17% to −0.60%) for Spo_2_ values of 91%.

**Table.**
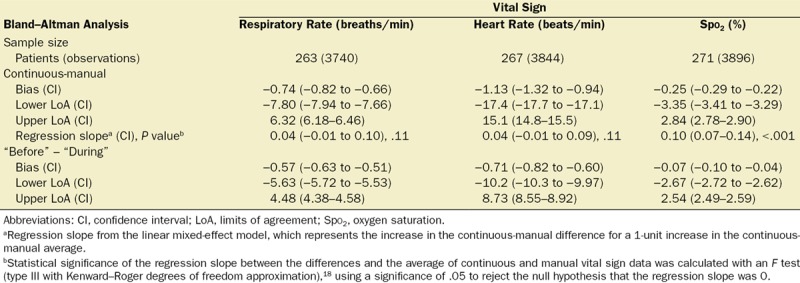
Results for the Bland–Altman Analysis Comparing Continuous Vital Sign Data to Manual Observations for Respiratory Rate, Heart Rate, and Oxygen Saturation

The bias (LoA) between the continuous and manual data was −0.74 (−7.80 to 6.32) breaths/min for respiratory rate, −1.13 (−17.4 to 15.1) beats/min for heart rate, and −0.25% (−3.35% to 2.84%) for Spo_2_ (Figure 3 and Table).

**Figure 4.**
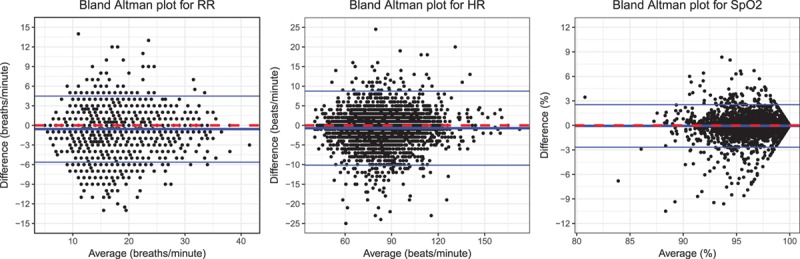
Bland–Altman plots for RR, HR, and Spo_2_ showing limits of agreement between continuous data sampled before the observation and continuous data sampled during the observation (“before” data – “during” data). Bias and limits of agreement are shown with blue lines, and a dashed red line shows the zero y-intercept. HR indicates heart rate; RR, respiratory rate; Spo_2_, oxygen saturation.

The bias (LoA) between continuous data before and during manual recording was −0.57 (−5.63 to 4.48) breaths/min for respiratory rate, −0.71 (−10.2 to 8.73) beats/min for heart rate, and −0.07% (−2.67% to 2.54%) for Spo_2_ (Figure 4 and Table). CIs (95%) for the bias and LoAs are reported in the Table.

Results for the sensitivity analysis of the primary assessment method for different window sizes (during the manual observation) are given in Supplemental Digital Content 1, Table 1, http://links.lww.com/AA/C522.

## DISCUSSION

We found no clinically relevant smoothing effect in postoperative care. If vital sign data were smoothed, then manual measurements would be recorded above abnormally low and below abnormally high continuous measurements, causing continuous-manual differences to be related to vital sign values. However, differences between manual and continuous heart rate and respiratory rate measurements were not related to the average values because the regression slope was not significantly different to 0. Differences between manual and continuous measurements of Spo_2_ were related to the measurement value. However, this relationship was clinically insignificant (Figure 3), with minor differences for low values of Spo_2_. For example, for Spo_2_ measurements of 91%, the model predicted a continuous-manual difference of −0.88% (−1.17% to −0.60%), which is within the measurement error of most pulse oximeters.^24^ The LoAs (95% CI) between continuously and manually recorded vital signs were large: 14.1 (13.8–14.4) breaths/min, 32.5 (31.9–33.3) heartbeats/min, and 6.2% (6.1%–6.3%) Spo_2_ between LoAs, suggesting that these recordings cannot be used interchangeably. We found no evidence of an arousal effect from the vital sign taker. The bias between continuous vital sign values recorded before and during manual observation was less than a single breath, heartbeat, or percentage Spo_2_.

Our sensitivity analysis showed that window size affected the relationship between continuous-manual differences and averages but not to a clinically meaningful extent (Supplemental Digital Content 1, Table 1, http://links.lww.com/AA/C522). As window size was reduced, lessening the effect of median filtering, the differences predicted by the model increased, although only by 1–2 heartbeats or breaths. Increasing the window size does not affect our findings, suggesting that the choice of a 5-minute window (also chosen by Reich et al^13^ and Taenzer et al^15^) appropriately removes artefact without increasing the sample size beyond what is plausible for bedside measurement. Notably, 4 previous studies found a smoothing effect using automated monitoring signals without temporal averaging, relying on manufacturer settings for artefact removal.^8,9,11,12^ Sapo et al^14^ demonstrated that Spo_2_ values <90% are associated with poor signal quality, suggesting that the effects in these studies may be due to clinicians correctly removing spurious values.

Further methodological differences may explain why our results contrast with the previous literature suggesting a smoothing effect.^8–10,12,13,22^ Four studies compared the magnitude and frequency of extreme values in manual and automated vital sign measurements where the automated measurement had higher measurement rates.^9,10,12,22^ Frequently sampled signals are more likely to capture transient extreme measurements than sparsely sampled signals, partly explaining the discrepancies found in these articles. Furthermore, 1 study compared manual and automated measures from different patient cohorts,^13^ while others only presented data from automated measurements^8^ or used simulated measurements from mannequins.^22^

In contrast to our Spo_2_ findings, Taenzer et al^15^ reported a difference of 6.5% between continuous and manual measurements of Spo_2_ <90% in general medical and postoperative wards. The method used continuous data to group Spo_2_ measurements >90% or <90%, thus comparing the differences between measurement sources to the values of the continuous source. Bland and Altman^25^ have shown that this process introduces a false correlation to the data. Hence, we compared the differences to the average values as recommended.^25,26^ For readers interested in this effect, we have replicated our analyses, comparing the difference against the continuous measure (and finding false correlations) in Supplemental Digital Content 2, Figure 1, http://links.lww.com/AA/C523.^25^

Manually recorded vital sign measurements varied widely from the continuous measurements. Respiratory rate is difficult to measure clinically,^2,3^ so the high variance in the differences between continuous and manual measurements is perhaps not unexpected. However, as early warning scores commonly include respiratory rate ranges between 2 scores of 4 breaths/min or less,^19,27^ these differences would commonly impact clinical care. The LoAs for heart rate and Spo_2_ were also wide and again would commonly result in different early warning scores. These results are important for those seeking to automate early warning scores,^28,29^ which have not been designed for use with continuous data, so patients would clearly alert differently.

Our study is limited by its single-center design because practices may vary between hospital wards and institutions. There may have been transcription errors in the value or timing of manual vital sign observations. This effect was minimized by double data entry and synchronizing observation times with computer-generated timestamps of blood pressure. One bedside monitoring provider was used in this study, so we could not assess differences between monitors. If we had found a relationship between the continuous-manual differences and averages, then this would have prevented exploration of whether the relationship could be explained by monitor inaccuracy, rather than clinician smoothing. Because there is no clinically significant relationship, this is not a significant issue for our findings. Our results are not influenced by undersampling because the measurement pairs of manual and continuous data sample the same physiology. The strengths of our article are the large dataset used in comparison to previous work and the analysis of 3 different vital signs.

We have provided evidence against the existence of a smoothing effect in postoperative care. However, this phenomenon may still exist in the context of anesthesia. It is possible that errors in vital sign documentation increase when clinicians record vital signs from memory after the measurement has been taken.^9,10^ This may be more pervasive in acute episodes during surgery and may not apply to postoperative wards, where nursing staff are available to measure and document vital signs simultaneously. Further research should investigate the smoothing effect during anesthesia, using the Bland–Altman method of assessing agreement between measurement sources, and comparing manually recorded measures to the median of a continuous window. If confirmed, the effects of the large differences between manually recorded and continuous vital sign measurements on early warning scores require investigation before the measurement and recording of these variables can be safely automated for early warning score computation.

## CONCLUSIONS

We found no evidence that a clinically significant smoothing effect exists for respiratory rate, heart rate, or Spo_2_ in postoperative care. We found no evidence of an arousal effect caused by the vital sign taker. Differences between manually recorded and continuous measures of respiratory rate, heart rate, and Spo_2_ were frequently large, suggesting that the methods cannot be used interchangeably.

## ACKNOWLEDGMENTS

The authors thank several individuals for their work on the Computer Alerting Monitoring System 2 study. Breda Lynch, Theresa Saunders, Sarah Vollam, and Deborah Evans conducted data collection; Jacqueline Birks undertook the statistical analysis and assisted in protocol development; and Julie Darbyshire assisted in proof reading. The authors would also like to thank Dr Lei Clifton for her work in preparing the dataset. Furthermore, the study could not have been undertaken without the support of the clinical staff on the upper gastrointestinal ward. The authors would like to thank the participants of the Computer Alerting Monitoring System 2 study for their commitment to research.

## DETAILS OF ACKNOWLEDGED INDIVIDUALS

Breda Lynch, Research Nurse, Oxford University Hospitals NHS Trust, Oxford, United Kingdom; Theresa Saunders, Research Nurse, Oxford University Hospitals NHS Trust, Oxford, United Kingdom; Deborah Evans, Research Nurse, Oxford University Hospitals NHS Trust, Oxford, United Kingdom; Sarah Vollam, Research Nurse, Oxford University Hospitals NHS Trust, Oxford, United Kingdom; Jacqueline Birks, MA, MSc, National Institute for Health Research–Oxford Biomedical Research Centre (NIHR–OXBRC) Senior Medical Statistician, Centre for Statistics in Medicine, Nuffield Department of Orthopaedics, Rheumatology and Musculoskeletal Sciences, University of Oxford, Oxford, United Kingdom; Julie Darbyshire, BA, MA, MSc, Critical Care Research Programme Manager, Oxford University Hospitals NHS Trust, Oxford, United Kingdom; and Dr Lei Clifton, BSc, MSc, PhD, Medical Statistician, Centre for Statistics in Medicine, Botnar Research Centre, Nuffield Department of Orthopaedics, Rheumatology and Musculoskeletal Sciences, University of Oxford, Oxford, United Kingdom.

## DISCLOSURES

**Name:** Hamish R. Tomlinson, BE (Hons).

**Contribution:** This author helped conduct the analysis and prepare the manuscript.

**Name:** Marco A. F. Pimentel, PhD.

**Contribution:** This author helped with conception and design of the analysis and drafting the manuscript.

**Name:** Stephen Gerry, MSc.

**Contribution:** This author helped with conception and design of the statistical methodology, and drafting the manuscript.

**Name:** David A. Clifton, PhD.

**Contribution:** This author helped with data acquisition and drafting the manuscript.

**Name:** Lionel Tarassenko, PhD.

**Contribution:** This author helped with conception and design of the analysis, data acquisition, and drafting the manuscript.

**Name:** Peter J. Watkinson, MBChB, MD.

**Contribution:** This author helped with conception and design of the analysis, data acquisition, and drafting the manuscript.

**This manuscript was handled by:** Nancy Borkowski, DBA, CPA, FACHE, FHFMA.

## Supplementary Material
