# Supplementary material for: Smoothing Effect in Vital Sign Recordings: Fact or Fiction? A Retrospective Cohort Analysis of Manual and Continuous Vital Sign Measurements to Assess Data Smoothing in Postoperative Care
Source: Anesth Analg. 2018 Aug 9;127(4):960–6. doi: 10.1213/ANE.0000000000003694 (PMC6135475; doi:10.1213/ANE.0000000000003694)
Supplement: Supplementary file 1 [file ane-127-0960-s001.docx]

| **Supplemental Table 1. Results for the sensitivity analysis comparing results for different window sizes (over which the “during” value is calculated). For window sizes from 1-10 minutes, we present the regression slope from the mixed effects model, the P-value, and the continuous-manual differences predicted at vital-sign values corresponding to low and high early warning score thresholds.** | | | | | |
| --- | --- | --- | --- | --- | --- |
|  |  |  |  | **Predicted difference ^a^ (C.I.)^b^** | |
| **Window size (minutes)** | | **Regression slope (C.I.)^b^** | **P -value^c^** | **Lower limit** | **Upper limit** |
| **Respiratory rate** | | |  | **(breaths/minute)** | |
|  | 1 | 0.17 (0.12, 0.21) | <0.001 | -1.9 (-2.3, -1.6) | 0.7 (0.3, 1.1) |
|  | 2 | 0.11 (0.07, 0.16) | <0.001 | -1.5 (-1.9, -1.2) | 0.2 (-0.2, 0.6) |
|  | 3 | 0.08 (0.03, 0.12) | 0.001 | -1.3 (-1.7, -0.9) | -0.1 (-0.5, 0.3) |
|  | 4 | 0.05 (0.00, 0.09) | 0.05 | -1.1 (-1.5, -0.7) | -0.4 (-0.8, 0.0) |
|  | 5 | 0.04 (-0.01, 0.10) | 0.10 | -1.1 (-1.6, -0.7) | -0.4 (-0.9, 0.0) |
|  | 6 | 0.04 (-0.02, 0.09) | 0.18 | -1.1 (-1.5, -0.7) | -0.5 (-1.0, -0.1) |
|  | 7 | 0.02 (-0.03, 0.07) | 0.42 | -1.0 (-1.5, -0.6) | -0.7 (-1.1, -0.2) |
|  | 8 | 0.01 (-0.04, 0.06) | 0.72 | -0.9 (-1.4, -0.5) | -0.8 (-1.3, -0.3) |
|  | 9 | 0.00 (-0.05, 0.05) | 0.95 | -0.9 (-1.3, -0.5) | -0.9 (-1.4, -0.4) |
|  | 10 | 0.00 (-0.06, 0.05) | 0.75 | -0.8 (-1.3, -0.4) | -1.0 (-1.5, -0.5) |
| **Heart rate** | | |  | **(beats/minute)** | |
|  | 1 | 0.07 (0.02, 0.12) | 0.003 | -3.9 (-5.7, -2.2) | 2.4 (-0.2, 5.0) |
|  | 2 | 0.06 (0.01, 0.10) | 0.02 | -3.4 (-5.2, -1.7) | 1.6 (-0.9, 4.2) |
|  | 3 | 0.05 (0.00, 0.10) | 0.04 | -3.2 (-5.0, -1.4) | 1.2 (-1.4, 3.8) |
|  | 4 | 0.04 (-0.01, 0.09) | 0.08 | -3.0 (-4.8, -1.2) | 0.8 (-1.8, 3.4) |
|  | 5 | 0.04 (-0.01, 0.09) | 0.10 | -2.9 (-4.7, -1.1) | 0.6 (-2.0, 3.3) |
|  | 6 | 0.04 (-0.01, 0.08) | 0.14 | -2.8 (-4.7, -1.0) | 0.4 (-2.3, 3.1) |
|  | 7 | 0.03 (-0.01, 0.08) | 0.16 | -2.8 (-4.6, -1.0) | 0.3 (-2.4, 2.9) |
|  | 8 | 0.03 (-0.02, 0.08) | 0.21 | -2.7 (-4.5, -0.9) | 0.1 (-2.6, 2.7) |
|  | 9 | 0.03 (-0.02, 0.08) | 0.25 | -2.6 (-4.4, -0.8) | -0.1 (-2.7, 2.5) |
|  | 10 | 0.02 (-0.02, 0.07) | 0.32 | -2.5 (-4.3, -0.7) | -0.3 (-2.9, 2.3) |
| **Oxygen Saturation** | | |  | **(%)** | |
|  | 1 | 0.15 (0.11, 0.19) | <0.001 | -1.3 (-1.6, -1.0) | NA |
|  | 2 | 0.12 (0.09, 0.16) | <0.001 | -1.0 (-1.3, -0.7) | NA |
|  | 3 | 0.11 (0.07, 0.14) | <0.001 | -0.9 (-1.2, -0.6) | NA |
|  | 4 | 0.11 (0.07, 0.15) | <0.001 | -0.9 (-1.2, -0.6) | NA |
|  | 5 | 0.10 (0.07, 0.14) | <0.001 | -0.9 (-1.2, -0.6) | NA |
|  | 6 | 0.10 (0.06, 0.13) | <0.001 | -0.8 (-1.1, -0.6) | NA |
|  | 7 | 0.11 (0.07, 0.14) | <0.001 | -0.9 (-1.2, -0.6) | NA |
|  | 8 | 0.10 (0.07, 0.14) | <0.001 | -0.9 (-1.2, -0.6) | NA |
|  | 9 | 0.10 (0.07, 0.14) | <0.001 | -0.9 (-1.2, -0.6) | NA |
|  | 10 | 0.10 (0.06, 0.13) | <0.001 | -0.9 (-1.1, -0.6) | NA |
| ^a^ The differences between the continuous and manual data that were predicted by the linear mixed effect model for (continuous-manual average) vital-sign values corresponding to low (left column) and high (right column) National Early Warning Score^[[1]](#footnote-1)^ thresholds (8 and 24 breaths/minute for respiratory rate, 40 and 130 beats/minute for heart rate, and 91% for oxygen saturation).  ^b^ 95% confidence interval.  ^c^ Statistical significance of the regression slope between the differences and the average of continuous and manual vital-sign data was calculated with an F-test (type III with Kenward-Roger degrees of freedom approximation)^18^, using a significance of 0.05 to reject the null hypothesis that the regression slope was 0. The regression slope represents the increase in the continuous-manual difference for a 1-unit increase in the continuous-manual average. | | | | | |

1. McGinley A, Pearse RM. A national early warning score for acutely ill patients. *Bmj*. 2012;345(aug08 1):e5310-e5310. doi:10.1136/bmj.e5310. [↑](#footnote-ref-1)
