# Supplementary figures and images for: Smoothing Effect in Vital Sign Recordings: Fact or Fiction? A Retrospective Cohort Analysis of Manual and Continuous Vital Sign Measurements to Assess Data Smoothing in Postoperative Care
Source: Anesth Analg. 2018 Aug 9;127(4):960–6. doi: 10.1213/ANE.0000000000003694 (PMC6135475; doi:10.1213/ANE.0000000000003694)

Incorrect Bland Altman for RR

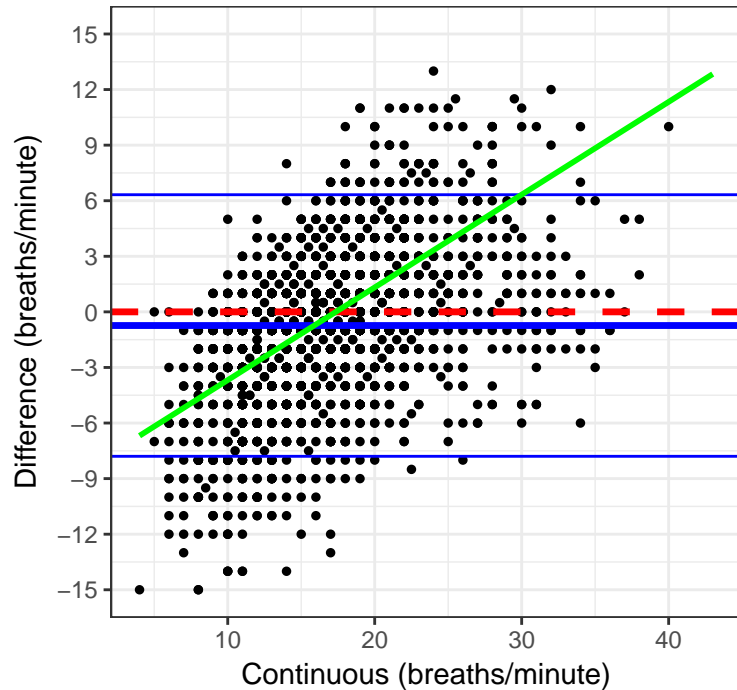

Incorrect Bland Altman for HR

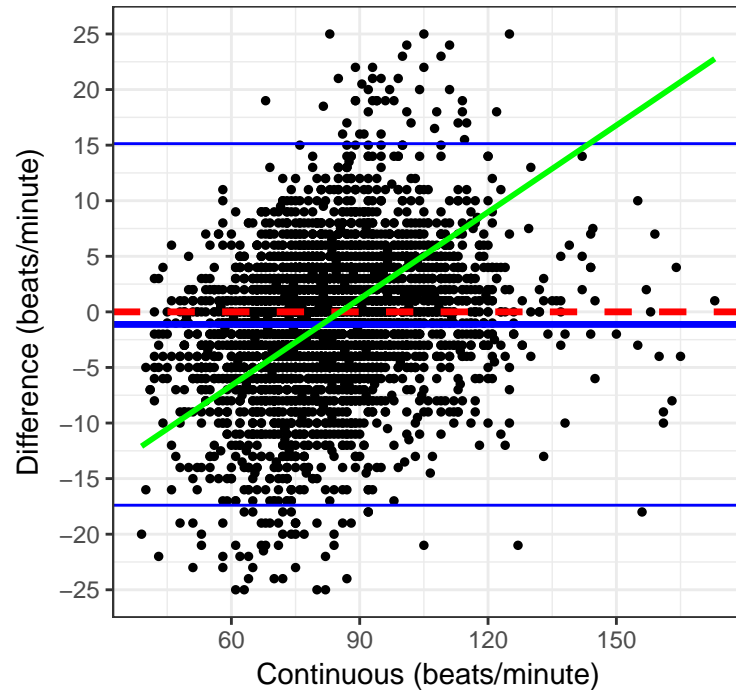

Incorrect Bland Altman for SpO2

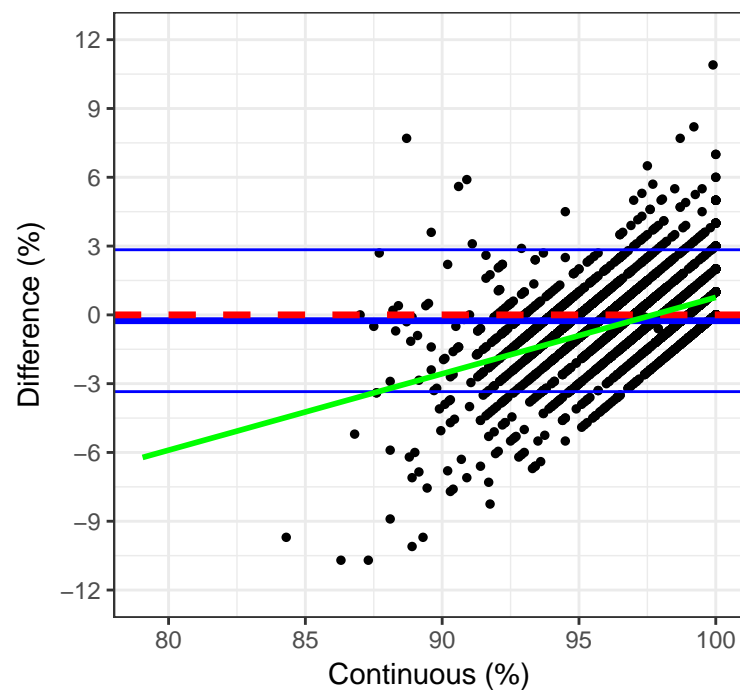

Supplement: Supplementary file 2 [file ane-127-0960-s002.pdf]
